# Occurrences on Board H. M. Hospital-Ship Gorgon, between the 18th of September, 1814, and the 8th of May, 1815

**Published:** 1818-01

**Authors:** William Boyd

**Affiliations:** at that time Surgeon of the Ship


### *Occurrences on Board H.M. Hospital-Ship Gorgon, between the* 18*th of September,* 1814, *and the* 8*th of May,* 1815. Reported by William Boyd, M.D. at that time Surgeon of the Ship.

THE Gorgon sailed from Plymouth-Sound on the 18th of September (accompanying an expedition, under the command of Major-General Keane, consisting of about five thousand men), with few sick on board; but shortly afterwards, we received some protracted cases of fever and chronic ophthalmia, from the troop-ships, with a few other trivial affections; all of which were soon restored to a state of health, and the major part discharged to rejoin their respective regiments, except one advanced case of pneumonia, which terminated fatally on the day after admission, probably from evacuations having been too long neglected in the primary stage of the disease.

The weather, during the earlier part of this month, proved rather unsettled, with heavy falls of rain, and occasional gales of wind; but towards the latter end of the month, it was fine, with increase of atmospherical temperature, as we approached the lower latitudes.

*October.* Nothing, in a medical point of view, occurred during this month. The weather was generally moderate and fine, with the wind northerly and westerly.

*November.* We reached Barbadoes on the 4th of the month: here some cases of inflammatory or ardent fever appeared amongst our own people, probably from their intemperate indulgence. This, together with increase of atmospherical temperature, and their having been employed on shore watering, exposed to solar influence, may reasonably be considered as the exciting causes of the disease,

The most prominent symptoms at the commencement of attack were shiverings, succeeded by universal lassitude, anxiety, head-ache (chiefly referred to the forehead and bottom of the orbits), shooting towards the temples; flushed, bloated countenance, with a watery suffused appearance of the eyes; pulse corded and firm; skin arid and hot; tongue white and moist, with red edges; thirst great; bowels unequal.

The means of cure adopted were copious abstraction of blood from the system at the commencement of the disease, purgatives, low diet, &c. with the subsequent exhibition of calomel, in the quantity of six, eight, or ten grains every four hours, either by itself or combined with small doses of opium and antimonial powder, according to circumstances, with an occasional purgative. This mode of treatment had the happiest effects in conducting the disease to a favourable issue.

I am not ignorant of the opinion, that calomel seldom, if ever, succeeds in affecting the salivary glands during febrile excitement; such, certainly, I have observed to be the case generally, although not universally; indeed, in the very worst cases of fever, the secretions into the alimentary canal, the diminished, nay, I may say, the total suspension of the absorbent system, will readily account for the non-specific action of mercury. Besides, we seldom or never observe much emaciation take place in fever until convalescence commences; at which period the absorbents seem to resume their action.

While we remained in Carlisle Bay, Barbadoes, the weather was fine, with light breezes from the eastward; but the nights were extremely sultry and close.

We sailed from Barbadoes on the 12th, and on the 14th received two cases of fever from the troop ships, both in the advanced stage. One of these died the following morning; but as no written statements were sent with them, I was unable to determine the means of cure adopted in the early stage of disease, the period at which evacuations would seldom fail in effecting the happiest results.

Blood abstracted from the temporal artery invariably afforded more immediate relief to the head, even in smaller quantity, than when drawn from the veins of the arm, although I am as yet undecided whether its effects are more permanent in producing a relaxed state of the vascular system.

Subsequently to this period, I was informed by Dr.

Morrell, apparently an intelligent gentleman, and surgeon to the marine hospital at New Orleans (who was detained on board of this ship for some time), that in New Orleans and its vicinity, the bilious remittent fever, in the months of June, July, August, and September, is the most distressing malady; and that dysentery is also the other most prevalent disease, particularly in autumn and winter. From the description he gave of the fever to me, it appears to be similar to the *inflammatory bilious fever* prevalent in the Mediterranean (with which I have had occasion to be conversant), and to be produced apparently from similar causes. Nothing else of professional importance occurred during this month: the weather generally proved fine, with increase of temperature, until we left Jamaica. Subsequent to that, a decrease of heat gradually took place, as we approached the coast of West Florida; so much so, as ultimately to descend to the freezing point, and occasionally lower.

*December.* After passing Jamaica and Cuba, we steered for the mouth of the Mississippi, and came to anchor off the *Isle de Chat*, in lat. 30 deg. 3 min. N. and long. 88 deg. 10 min. W. At this time, the boats of the fleet, manned and armed, were sent to attack the enemy's flotilla of five gun-boats, lying at *Malheureux* Islands (Gulf of Mexico), some distance from New Orleans; and they succeeded in capturing this force, on the 14th, with considerable loss on both sides.

On the 15th, we received two officers, eleven seamen and marines, and eleven prisoners of war; and on the 21st, two seamen and seven prisoners of war, the major part of whom were severely wounded with gun-shot, splinters, and buck-shot.

Four amputations became necessary amongst the above injuries; namely, one at the shoulder joint; one of the left thigh, at the trochanter minor; and two of the right thigh, about two inches below the trochanter minor; all originating from severe wounds, accompanied with a fractured, or rather shattered, state of the bones.

I have only to regret, that, upon the receival of these cases, the state of tumefaction, inflammation, &c. (no doubt much increased by delay, from the distance they had been conveyed in boats previous to reaching this ship), precluded immediate amputation; which, I am strongly disposed to think, would have been the only means of removing the source of irritation, preventing the subsequent inflammation, and consequently the profuse suppuration, always attendant upon injuries of such magnitude.

The case of amputation at the shoulder joint originated from a gun-shot wound above the middle part of the arm. The ball penetrated at the outer edge of the biceps muscle, and made its exit, in an oblique direction, about four inches below the acromion process of the scapula, a little below the outer and inferior edge of the deltoid muscle; shattered the bone, and injured the brachial artery, so as to produce profuse hæmorrhage on the sixth day subsequent to the infliction of the wound. At this period, though there still existed a degree of inflammation, circumstances rendered the removal of the arm immediately necessary; and it was followed by great alleviation of the sufferings of the patient. On dissection of the arm (after its removal), nearly one half of the diameter of the brachial artery was found carried away and the bone splintered, or rather pounded, as high up as the cervix of the os humeri. This patient did well.

The three cases of amputation, high in the thigh, I am sorry to state, terminated fatally, seemingly from the powers of life being inadequate to support the loss, or produce the requisite degree of action in the stumps, so absolutely necessary for the re-union of parts.

On the 18th, we received some cases of dysentery from the army, and one seaman (frost-bitten) with loss of his toes.

On the 23d, our troops had a skirmish with the enemy, shortly after landing in Louisiana; and on the 31st we received a large proportion of wounded. Most of the cases were attended with considerable inflammation, from their not having been regularly dressed, nor other appropriate means used previous to their being sent on board this ship: all of the above wounds were from grape, musket, and buck shot; several of them very severe, particularly about the face and jaws.

*January.* One amputation of the arm was necessary on the 1st, in consequence of a gun-shot wound of the elbow joint, attended with fracture of both condyles of the humerus, and injury of the ligaments, &c. On the 6th, we received some dysenteric and frost-bitten cases from the army; and on the 12th and 13th, a great number of wounded troops; in consequence of an attempt having been made, on the 8th, to storm the enemy's entrenchments before New Orleans, in which we, unfortunately, were repulsed with great loss.

From the wounded not having been regularly attended to, from the 8th until the period of their gaining this ship, many of them were suffering severely from the effects of inflammation, fatigue, and cold: one of them died during the night, from mortification of the thigh and leg, induced by a gun-shot wound (or rather splinter-wound) of the calf of the leg. We received also several cases of mortification of the feet and toes, hands and fingers; chiefly from the native West India regiment, who evidently suffered greatly from the sedative effects of cold. Some of these cases were so severe as to be followed by loss of the hands and feet, and others of toes, fingers, and part of the feet and hands. As the disease was generally in an advanced state previous to admission, I found stimulating local applications, with the internal exhibition of opium and wine, afford the only relief.

One case of sympathetic tetanus occurred in a man of colour, afflicted with mortification of the right foot and toes, which terminated fatally on the third day after the attack, notwithstanding his having taken four ounces of tinct. opii, and about two pints of rum, in thirty-two hours, made into punch, without producing any permanent relief. It is true, the spasms seemed lessened in a slight degree, but this relief was of no permanent duration; indeed, the dysphagic symptoms became so distressing latterly, as to prevent his swallowing almost any thing. The pulse, at the commencement of the attack, was small, soft, and pretty regular; but, after the exhibition of the tinct. opii and spirits, it became fuller, and, during the last twelve hours of his illness, it was small, quick, and irregular, with profuse perspiration.

Several cases of tetanus occurred amongst the wounded in the squadron, although, with the exception of the above, not an individual treated on board of this ship exhibited the slightest appearance of this most formidable disease. This, I am strongly inclined to think, may be attributed, in a great measure, to the bowels of our wounded having been kept open during the whole period of their confinement; for I have long been of opinion, that a torpid state of the bowels predisposed to, and was a usual concomitant of, both traumatic and idiopathic tetanus.

The means of cure I found to succeed with the wounded, were the abstraction of blood from the system (where inflammation, pain, and tension, presented themselves), laxatives, low diet, &c. with the constant application of cold to the parts locally injured; warm applications I found invariably induced a relaxed state of the parts, with profuse suppuration and great pain: whereas the cold universally afforded relief, with diminution of morbid heat, and mild suppuration. It is but right to observe, that warm applications, where tension prevailed (without morbid heat), and disposition to gangrene, seemed to claim the preference; this, with the internal exhibition of opiates, combined with antimonials, constituted our general or constitutional treatment.

The weather, during this month, proved extremely cold, with great decrease of atmospherical temperature; so as to be productive of dysentery, cynanche tonsillaris, coughs, &c. especially about the latter part of the month.

*February.* The weather continued unsettled during the early part of this month; but towards the latter end it became more moderate, with increase of heat.

We continued in the Gulf of Mexico, off the *Isle de Chat*, until the 17th, when we sailed for *Dauphin Island.*

Dysentery became the most prevalent disease during this month; some cases of scorbutus also presented themselves; as also some cases of debility from fatigue and want of the necessaries of life.

The most prominent dysenteric symptoms were, prostration of strength, head-ache, nausea, and vomiting of greenish bilious matter, accompanied with severe tormina and frequent small bloody stools; pulse generally quick, and sometimes full; skin dry and warm; tongue white and dry in some, and in others moist and clammy; thirst generally great. I have no doubt that most of the recent cases which came under my care were attended with a degree of inflammation of the mucous membrane of the intestinal canal, with determination to the liver, accompanied with a diseased biliary secretion.

The means of cure I found most successful in arresting the progress of the disease were, the detraction of blood from the system at the commencement of attack, when excitement seemed to render it necessary; and the subsequent exhibition of calomel, in doses of 10, 15, or 20 grains, combined sometimes with opium and antimonial powder, and repeated twice or thrice a day; with the occasional interposition of a laxative, or of the warm bath, when the irritation of stomach and pungent dryness of skin prevailed. These had the happiest effects in conducting the disease to a favourable termination; indeed, every recent case invariably yielded, as soon as the specific effects of mercury became visible. Thereafter, a slight infusion of some vegetable tonic bitter was usually given during convalescence, with occasionally 10 or 12 grains of the compound powder of ipecacuanha at bed-time.

A strong disposition to slough occurred amongst several of our wounded, demanding the most rigid attention to separation, ventilation, and cleanliness; together with the local employment of cold lotions, the internal exhibition of laxatives, and the enforcement of the antiphlogistic regimen, during the progress of gangrenous inflammation or sloughing. I have often felt how much more it is within the power of our art to reduce increased excitement, and to check over action, than to rouse new energy, or suspend the tendency to decomposition and death of parts, when once that tendency is fairly established; and I am persuaded, that many a patient has fallen a victim to the premature exhibition of bark, wine, opium, and brandy, when a dark-coloured speck has appeared on the surface of a wound, or of an inflamed tumour. In such cases, therefore, in my opinion, the primary and more early treatment ought to be invariably, and *in all its parts,* anti-inflammatory.

One case of fever, with great determination to the lungs and brain, terminated fatally during this month; most probably from the disease having been neglected at the commencement, in consequence of the patient's being employed up the river in boats, during the primary stage of his illness.

Another fatal case was one of mortification of the feet and hands; the former were removed above the ankles, the latter at the wrists: this man appeared doing well until a few hours before dissolution, when the vital powers seemed to subside rapidly! Generally, I have observed such to be the case in the instances of death occurring amongst the native West India soldiers.

On the 23d, we sailed from *Dauphin Island* for Havannah; but, previous to our leaving the former, we received from the squadron several wounded soldiers: likewise several cases of sloughing ulcers, scorbutus, and dysentery, all of considerable standing, and attended with great irritation and general prostration of the powers of life. The dysenteric cases were mostly combined with scorbutus, and the whole in an advanced state of disease; this rendered the treatment very different to that before stated, in the recent cases of the complaint: that is, calomel, given in any proportion, seldom or ever produced any sensible effect. This, I think, may reasonably be attributed to the inertness of the lymphatic system. In such cases, I found a combination of opium and antimonial powder, in the proportion of one grain of the former to three of the latter, given three times in the course of the day, or eight grains of the compound powder of ipecacuanha, afford the greatest solace from suffering.

Mercurial inunctions of the abdomen and hepatic region were had recourse to, in several instances, with evidently good effects; particularly when the irritable state of the *primæ viæ* prohibited the internal use of calomel. But from many of them being in such an extenuated condition before their admission, little relief could be afforded by medicine: in short, supporting the vital functions previously to dissolution, seemed all that could possibly be effected.

The prominent symptoms of those afflicted with scurvy were general prostration of strength, pain in the loins, bloated, anxious countenance, listlessness, and disinclination to motion, with a spongy, jagged appearance of the gums (which bled upon the slightest touch); and, in several cases, considerable pain and ulceration round the alveolar processes of the teeth, with œdema and lividity of the lower extremities, hardness and contraction of the flexor muscles of the legs and hams. These symptoms were commonly attended with oppressed breathing, particularly towards evening, and during the night. The major part affected had been exposed to great fatigue, cold and wet (with no vegetable diet for a considerable time), during the attempt upon New Orleans, in landing troops, carrying provisions, and performing other laborious duties subservient to the army. At the same time, they had used a considerable quantity of spirituous liquors, served out in an undiluted state, and salt provisions, without any vegetable substance whatever. This combination of circumstances could scarcely fail to induce a general scorbutic diathesis.

Our wounded cases were all clean, and apparently doing well, although a few of them had sloughed previously; but the fresh cases of ulcer in a sloughing state, notwithstanding every attention, spread the contagion amongst a few of our clean sores; yet, by attention to dressing, separation, &c. (as before observed), their action was soon reconverted to that of a healthy sore. The internal means were laxatives, with lemonade to drink, and the topical application of stimulants of various kinds; however, I

must observe, that of all the external applications to ulcers, when in a sloughing state, a strong solution of the sulphate of alum seemed evidently to excel, in destroying the fœtor, concentrating the slough, and producing a healthy discharge, with subsequent sound granulations. As we had no fresh meat, at least little, comparatively speaking (though the ship's boats caught some fish occasionally, from which the sick were always served in preference to the rest), without any supply of fresh vegetable matter; nay, even without a sufficiency of lemon-juice, our resources were naturally much contracted: nevertheless, by substituting flour, sugar, &c. in lieu of meat, the evil was in some measure obviated.

*March.* We arrived at Havannah on the 1st of this month, and were liberally supplied with fresh vegetables; namely, pompions, yams, cabbages, oranges, &c. and fresh animal food: these produced a very sensible amendment amongst our wounded and scorbutic cases; and as we experienced considerable increase of atmospherical temperature, and were now able to keep the ship perfectly ventilated, the tendency to disease was much lessened.

On the 7th, we received several men from the *Seahorse*, *Cydnus*, *Calliope*, and *Bedford*; most of whom were afflicted with wounds, ulcers, and chronic dysentery.

On the 9th we put to sea, but our crowded state, and the ulcers remaining on board, still produced and kept up a disposition to sloughing. As the weather proved unsettled, with falls of rain, and severe storms of wind, whilst in the Gulf of Florida, we could not sufficiently air the hospital-deck, which operated greatly against our endeavours to keep the sores clean. Notwithstanding this, our wounds continued progressively improving under the plentiful supply of vegetable matter received at Havannah; and all those afflicted with the scorbutic diathesis were completely restored to their former state of health. The only troublesome affections were bowel complaints, which attacked many of those recovering from their wounds; however, they readily yielded to mercury, combined with antimony and a small proportion of opium.

On the 25th, the ship arrived at Bermuda, in a tolerable state of health, having only two sloughing ulcers on board, with some obstinate protracted cases of dysentery, which we received in an advanced stage of disease: some of them terminated fatally, baffling every means of cure.

The weather was moderate till the 10th, when we had strong gales for some time; but it became more moderate towards the latter end of the month, the wind being generally easterly and southerly.

*April.* We sailed from Bermudas on the 7th, with a few bowel complaints on board, probably originating from atmospherical vicissitudes, assisted, on the part of the people, by indulgence in spirituous liquors (evidently of bad quality), whilst on shore watering, and performing the other necessary duties of the ship. The water we received at this place was brackish; and this also may have tended to produce some unusual irritation of the intestinal canal. I am unwilling, however, to consider the water as the only exciting cause.

We had very heavy gales of wind for ten or twelve days after leaving Bermuda, which prevented the hospital from being ventilated. Then, owing to the wet and crowded state of the ship, some of the wounds began to exhibit a disposition to slough; but fortunately the weather became fine, so as to admit the thorough perflation of the lower deck, and other parts of the ship. As we had been liberally supplied with *preserved meat*, for the use of the sick, by order of our humane Commander in Chief, the Honourable Sir A. Cochrane, almost all were soon restored to a state of health.

*May.* On our arrival at Spithead, on the 5th of the month, there was little or no disease on board; indeed, such as had been unwell were convalescent; and the greater part of the wounded were nearly well, so that only eighteen cases required to be sent to Haslar on the 7th, and those were trivial. The others, belonging to the army, were landed at Portsmouth on the 8th, and sent to Hilsea Barrack: the occurrences in the hospital-ship became of no moment subsequent to this period.

The preceding is a concise Report of professional events on board this ship during the period which it embraces. The varieties of climate and vicissitudes of temperature in our frequent and sudden transitions from cold to heat, and from heat again to cold, must necessarily have been great; but my arduous professional duties, added to the other avocations that a ship of this sort imposes, prevented me from being able (as I could have wished) to give a more full and correct detail, both in a medical and meteorological point of view, of the various circumstances that came under my observation at this interesting crisis.

W. BOYD.

